# Triamcinolone and Bevacizumab as Adjunctive Therapies to Panretinal Photocoagulation for Proliferative Diabetic Retinopathy

**DOI:** 10.5402/2012/267643

**Published:** 2012-10-15

**Authors:** F. Lopez-Lopez, F. Gomez-Ulla, M. J. Rodriguez-Cid, L. Arias

**Affiliations:** ^1^Ophthalmology Department, University Hospital Complex, Ramon Baltar s/n, 15706 Santiago de Compostela, Spain; ^2^Gomez-Ulla Institute of Ophthalmology, Avenida de las Burgas 2, HNS de la Esperanza, 15705 Santiago de Compostela, Spain; ^3^Ophthalmology Department, Bellvitge University Hospital, Avenida Granvia s/n, 08907 L'Hospitalet de Llobregat, Barcelona, Spain

## Abstract

*Purpose*. To evaluate efficacy of intravitreal triamcinolone (IVT) and bevacizumab (IVB) as adjunctive treatments to panretinal photocoagulation (PRP) in proliferative diabetic retinopathy (PDR). *Methods*. In 60 eyes of 45 patients with PDR, PRP (PRP group), PRP with IVT (IVT group), or PRP with IVB (IVB group) was performed. Regression of new vessels (NV), changes in best-corrected visual acuity (BCVA), central macular thickness (CMT), and contrast sensitivity at 1,2, and 6 months were evaluated. *Results*. Initial mean numbers of active NV and BCVA were 3.45 and 67.35 in the PRP group, 4.35 and 76.65 in the IVT group, and 4.79 and 75.53 in the IVB group. At the 6-month follow-up, numbers of active NV were 2.5 (*P* = 0.064), 1.11 (*P* = 0.000), and 1.11 (*P* = 0.002), and there was a mean loss of 2,6 (*P* = 0.055), 3.9 (*P* = 0.011), and 0.9 letters (*P* = 0.628) in the PRP, IVT, and IVB groups, respectively. Changes in CMT in the PRP and IVT groups were not significant, but significantly increased in the IVB group (*P* = 0.032). Contrast sensitivity remained stable in PRP and IVB groups and slightly decreased in IVT group. *Conclusions*. Adjunctive use of both triamcinolone and bevacizumab with PRP lead to a greater reduction of active NV than PRP alone in PDR, although no differences were seen between the two of them.

## 1. Introduction

Diabetic retinopathy is one of the leading causes of blindness in the developed world, particularly in patients aged 20 to 74 years [1]. Its estimated prevalence is 70.4% in patients with type 1 diabetes and 54.4% in patients with type 2 diabetes [2, 3]. 

Microvascular occlusion in the eye caused by the glycation of blood vessel proteins results in local hypoperfusion that leads to retinal ischaemia. Proliferative diabetic retinopathy (PDR) occurs in response to the ischaemia-mediated release of vascular endothelial growth factor (VEGF) into the vitreous cavity [4–6]. It has been shown that retinal neovascularisation is a significant risk factor for severe vision loss in diabetic patients [4].

The Diabetic Retinopathy Study demonstrated that scatter laser panretinal photocoagulation (PRP) reduced the risk of severe vision loss by at least 50% in patients with high-risk PDR compared to a control group [7]. Currently, PRP is the principal therapy for sight-threatening PDR except for patients with a history of extensive vitreous haemorrhaging, which is a contraindication to laser photocoagulation. 

Intravitreal injections of several drugs in combination with PRP have been shown to achieve more favourable therapeutic outcomes than PRP alone. The adjunctive use of triamcinolone with PRP leads to a higher rate of new vessel regression and reduced macular oedema compared to PRP alone, based on several studies [8–10]. Drug-related side effects, such as cataract progression and secondary glaucoma, were commonly observed in the injected patients. 

The intravitreal injection of the antivascular endothelial growth factor (VEGF) antibody drug, bevacizumab, was tested to determine whether similar beneficial results of new vessel regression and reduced macular oedema can be achieved without the side effects. The first published studies reported promising short-term outcomes [11, 12], and comparative trials confirmed these results [13–15]. 

The major purpose of this prospective, controlled study was to evaluate the effectiveness of intravitreal triamcinolone (IVT) or intravitreal bevacizumab (IVB) combined with PRP on retinal neovascularisation and visual acuity changes compared with PRP alone in patients with PDR.

## 2. Methods

### 2.1. Patient Eligibility

We recruited consecutive patients who visited the Medical Retina Unit of the University Hospital Complex of Santiago de Compostela and met the inclusion criteria. 

We excluded from the study patients with the following characteristics: previous panretinal or focal/grid photocoagulation, signs of vitreomacular traction (either on optical coherence tomography (OCT) or biomicroscopy), a history of cataract extraction or lens implantation within the previous 6 months, significant media opacities or a history of glaucoma or ocular hypertension. Patients with high glycated haemoglobin (HbA1c) levels or high blood pressure were not excluded.

The study protocol complied with the Helsinki declaration and was reviewed and approved by the Bioethics Committee of the University of Santiago de Compostela. The Spanish Ministry of Health approved the use of bevacizumab in each patient. Written informed consent was obtained from all patients after a clear explanation of the nature of the intervention was provided. The informed consent clearly stated that patients were involved in a scientific investigation.

### 2.2. Preoperative Examination

To determine the PDR severity, the same examiners (F.L.L. and F.G.U.) graded the patients' retinopathy levels based on slit-lamp biomicroscopy, retinography, and fluorescein angiography (FA), according to the modified retinopathy severity scale of the ETDRS Research Group [20].

Baseline data included age, sex, type, and duration of diabetes mellitus, blood pressure measurements, HbA1c levels and serum cholesterol and triglyceride levels.

Patients underwent a clinical examination that included refraction, and best-corrected visual acuity (BCVA) was measured according to a standardised refraction protocol using a retroilluminated Lighthouse for the Blind distance visual acuity test chart (using modified ETDRS charts 1, 2, and R) [21]. The contrast sensitivity was measured using the Pelli-Robson test (retroilluminated Lighthouse charts), and lenticular status was classified by comparing stereoscopic lens photographs based on the Lens Opacities Classification System III [22]. Tonometry was measured with a Perkins applanation tonometer, and fundus examinations, retinographies, and FA were performed using digital angiograms that were captured with a Topcon retinal camera (model TRC-50IX; Topcon, Tokyo, Japan) using IMAGEnet software 2.5. The foveal thickness was measured to calculate the average thickness in the central ring (1000 *μ*m in diameter) using a commercially available fast macular thickness scanner from Stratus OCT (Carl Zeiss Meditec, Dublin, Ireland). 

### 2.3. Experimental Design

This prospective, comparative, interventional pilot study included 60 eyes. The first 40 eyes were randomised (1 : 1) to receive either standard PRP alone (the PRP group) or PRP plus one intravitreal injection of triamcinolone (the IVT group). Due to the popularity of anti-VEGF agents, we created an additional group (the IVB group) that consisted of the next 20 consecutive eyes in patients who met the inclusion criteria; PRP plus three intravitreal injections of bevacizumab one month apart were administered in these eyes.

Either IVT or the first dose of IVB was injected immediately after the first laser treatment. No focal/grid laser was used before or during the follow-up period.

### 2.4. Intravitreal Injections

After the first laser treatment was completed, each eye was prepared using prophylactic antibiotic drops and 5% povidone iodine. Using a 30-gauge needle, the injections were administered 4 mm posterior to the corneal limbus through the inferior pars plana. An eyelid speculum was used to stabilise the eyelids. Immediately after each injection, the patient was examined using indirect ophthalmoscopy to observe the circulation in the central retinal artery, and the patient's light perception was verified. Each patient was instructed to apply antibiotic (ciprofloxacin) eye drops four times per day for seven days following the injection.

Eyes that were assigned to the IVT group were treated with 4 mg of triamcinolone acetonide (0.1 mL per injection) directly from the vial (Trigon depot^®^, Bristol-Myers Squibb, s.l., Madrid, Spain), and the IVB-group eyes were treated with 1.25 mg of bevacizumab (Avastin^®^, Genentech Inc., San Francisco, CA, USA) in 0.05 mL per injection. All eyes were injected under topical anaesthesia by the same surgeon (F.L.L.).

### 2.5. Scatter PRP

Scatter PRP was performed under topical anaesthesia in 3 sessions one week apart using a 532-nm green laser (IRIS Medical OcuLight GL) and a Mainster Wide Field lens. The inferior and the temporal retina were treated in the first session, and nasal and superior retina were treated in the second and third sessions, respectively. The spot size used was 300 *μ*m, the exposure time was 0.1 sec, and the power was adjusted to produce a grey-white lesion. All eyes were treated by the same ophthalmologist (F.L.L.).

Two months after the laser treatment, FA was performed. Any residual ischaemic or untreated areas were addressed with an additional laser treatment. 

### 2.6. Outcome Measures

The main outcome measures included retinal neovascular regression, which was defined as the complete disappearance of vitreous leakage from new vessels in the disk (NVD) and new vessels elsewhere (NVE) during any phase of the fluorescein angiogram, and BCVA changes measured using the ETDRS charts at one, two, and six months after the treatment.

Secondary endpoints included the change in the central macular thickness (CMT) based on OCT, changes in the contrast sensitivity, changes in the intraocular pressure, and the incidence of moderate or severe adverse effects at the same time-point.

### 2.7. Follow-Up Examinations

Patients were scheduled for follow-up examinations at one, two, and six months after the treatment. The same procedures that were performed at baseline were repeated at the two- and six-month follow-up visits (e.g., BCVA assessment, complete ophthalmic examination, photography, and FA). Systemic and local adverse events, including changes in the intraocular pressure and lens status, were monitored throughout the study.

### 2.8. Statistical Analysis

Normal distributions of the data were demonstrated using the Kolmogorov-Smirnov test, and the Student's *t*-test and ANOVA were used in the statistical analysis. Quantitative data are expressed as mean ± standard deviation (SD). The level of significance was set at *P* < 0.05. All analyses were performed using SPSS 16.0 for MAC.

## 3. Results

Between December 2005 and November 2007, 60 eyes from 45 patients were included in this study. Based on the clinical examination and FA, all of the eyes presented with PDR with or without clinically significant macular oedema. The mean (SD) age was 54.74 (13.85) years (range: 22–76 years). There were 11 female patients and 34 male patients. 9 patients had type 1 diabetes, and 36 had type 2 diabetes. All but 2 eyes were phakic (1 in the PRP and 1 in the IVT groups). The clinical characteristics of the eyes before treatment are summarised in Table 1. Note that no statistical differences were observed in the baseline characteristics between the PRP, IVT, and IVB groups.

There were no differences in the mean (SD) number of laser spots performed in each group (2742.21 (479.19), 2725.53 (524.32), and 2968.5 (469.01) in the PRP, IVT, and IVB groups, resp.) or the number of eyes that were retreated.

### 3.1. Primary Outcomes

**Figure 1:**
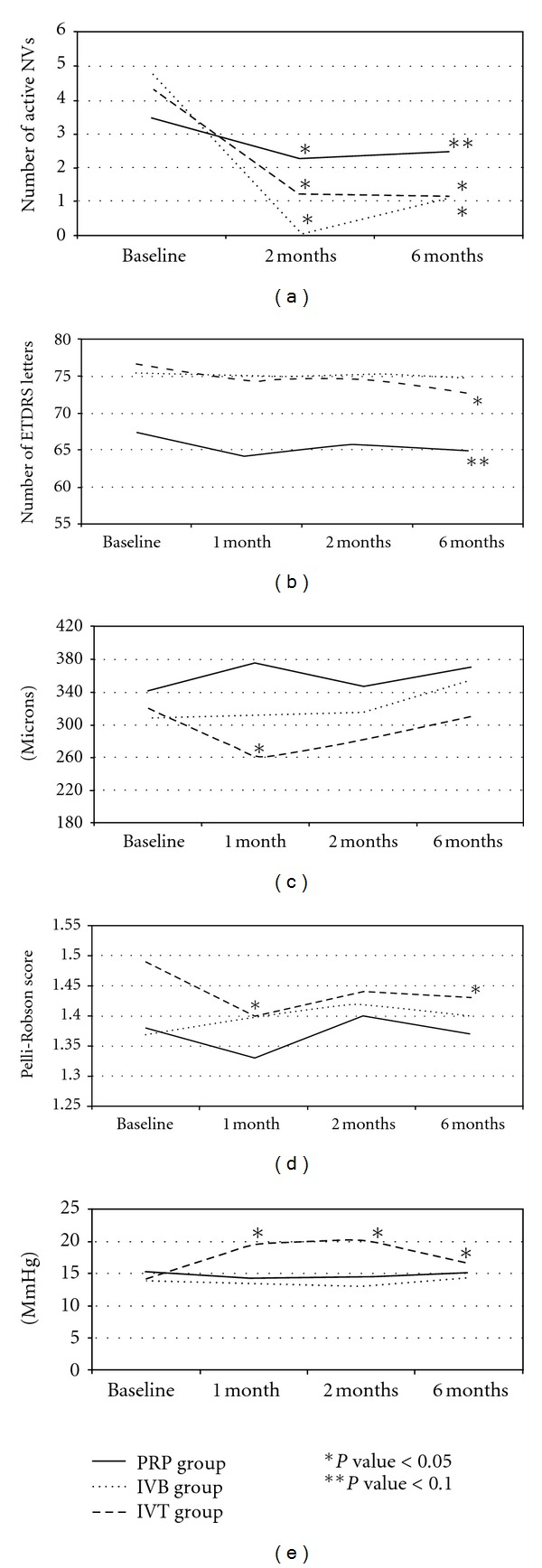
(a) Changes in the activity of the new vessels in the three groups during the follow-up period, based on the diffusion observed on fluorescein angiography. (b) Changes in the visual acuity (number of ETDRS letters) in the three groups during the follow-up period. (c) Changes in the central macular thickness (microns) in the three groups after treatments. (d) Changes in the contrast sensitivity (Pelli-Robson score) in the three groups during the follow-up period. (e) Intraocular pressure changes (mmHg) in the three groups during the follow-up period. The Student's *t*-test was used to compare the changes in the outcomes to the baseline values for the three groups. *P  value < 0.05 and **P  value < 0.10.

Number of active new vessels in the eyes that were treated with PRP alone decreased from a mean of 3.45 at baseline to 2.28 (*P* = 0.049) and 2.5 (*P* = 0.064) at the two-, and six-month follow-up visits, respectively. IVT plus PRP reduced the number of active new vessels from a mean of 4.35 at baseline to 1.25 (*P* = 0.000) and 1.11 (*P* = 0.000) at the two- and six-month follow-up visits, respectively. Active new vessels in the IVB group decreased from a mean of 4.79 at baseline to 0.05 (*P* = 0.000) and 1.11 (*P* = 0.002) at the two- and six-month follow-up visits, respectively (Table 2 and Figure 1(a)).

 The combination of triamcinolone and PRP significantly reduced the number of new vessels at the six-month follow-up visit compared with PRP alone (*P* = 0.024 using the Student's *t*-test; normal distribution of the data was demonstrated using the Kolmogorov-Smirnov test). The combination of bevacizumab and PRP also produced better results than PRP alone (*P* = 0.001 at two months and *P* = 0.021 at six months; in both cases, the Student's *t*-test was used to compare the data after a normal distribution was demonstrated using the Kolmogorov-Smirnov test). The combination of bevacizumab and PRP was also more effective than IVT plus PRP at the two-month follow-up visit (*P* = 0.013), but no differences were observed at the six-month follow-up period between these two groups (*P* = 1.0).

According to the modified retinopathy severity scale from the ETDRS Research Group [20], retinopathy was stable (no progression) in 85% of the eyes in the PRP group after PRP was performed compared with 100% of the eyes in both the IVT and IVB groups although these differences were not significant (*P* = 0.680 using ANOVA). A reduction in the severity of the retinopathy (a decrease of at least one level) was achieved in 53% of the eyes in the PRP group, which was a lower percentage than that of the other groups; a reduction in the severity of the retinopathy was achieved in 65% of the eyes in the IVT group, and in 79% of the eyes in the IVB group. Again, these differences were not significant (*P* = 0.505, using ANOVA).

In eyes with high-risk PDR, PRP induced regression in 2/5 eyes; the PRP plus IVT combination reduced the severity of the retinopathy in all cases (5/5), and the PRP plus BIV combination reduced the severity in all cases except one (7/8). Furthermore, two cases from the PRP group involved vitreous haemorrhages during the follow-up period, despite the treatment. The severity of the diabetic retinopathy in the different groups before and after the treatments is summarised in Table 3.

Visual acuity remained stable (a gain or loss of no more than 5 ETDRS letters) in all groups during the follow-up period. Patients who received PRP alone experienced a mean loss of 2.6 ETDRS letters, while the IVB and IVT groups experienced a mean loss of 0.9 letters and 3.9 letters, respectively (Figure 1). Visual acuity changes were not significant in any group at any time point, except in the IVT group at the six-month follow-up visit (*P* = 0.011) (Table 2 and Figure 1(b)). There were no significant differences in BCVA between the groups during the follow-up period.

Five eyes PRP-group suffered a vision loss of two or more ETDRS lines (in two cases, there was a four-line loss). Two eyes in both the IVT and IVB groups suffered a loss of two or more lines (one case in the IVT group experienced a four-line loss).

### 3.2. Secondary Outcomes

The CMTs changes in the control group were not significant during the follow-up period (*P* = 0.124, *P* = 0.402 and *P* = 0.129 at the one-, two- and six-month follow-up visits, resp.). In the IVB group, the CMT remained stable while eyes were being injected (*P* = 0.902 and *P* = 0.671 at one and two months, resp.) but significantly increased by a mean of 45.3 microns at the six-month follow-up visit compared to baseline (*P* = 0.032). In contrast, the IVT group experienced a significant CMT reduction at the one-month follow-up visit (*P* = 0.02), and although the CMT was reduced compared to the baseline, the differences were not significant at the two- and six-month follow-up visits (*P* = 0.134 and *P* = 0.857, resp.) (Table 2).

The IVT group showed a significant CMT reduction compared to PRP alone at both one and two months after treatment (*P* = 0.002 and *P* = 0.032, resp.), and although there were no statistical differences at six months (*P* = 0.11), the confidence intervals showed a tendency towards significance (CI:−15.2; +133.58). The IVB group failed to reduce the CMT compared with PRP alone at any point during the follow-up period, and no significant differences were observed between the IVT and IVB groups, except at the one-month follow-up visit (*P* = 0.032) (Figure 1(c)).

The mean baseline contrast sensitivity (using the Pelli-Robson score) was 1.38 (0.38) in the PRP group, 1.49 (0.20) in the IVT group and 1.37(0.25) in the IVB group. Changes in the contrast sensitivity were not significant during the follow-up period, except in the IVT group, which showed a decrease in the sensitivity at six months (*P* = 0.034). The statistical analysis did not reveal significant changes among the groups (Figure 1(d)).

Changes in the intraocular pressure (IOP) were not significant in the PRP and IBV groups during the follow-up period, but the eyes in the IVT group showed a significant increase in the IOP compared to the other two groups. At the one-month follow-up visit, the IOP in the IVT eyes was 5.6 mmHg higher than at baseline (*P* = 0.001), and at the two- and six-month follow-up visits, the IOPs were 6.1 and 2.6 mmHg higher than at baseline, respectively (*P* = 0.000 and *P* = 0.027). IOP changes are summarised in Figure 1(e).

Three eyes in the PRP group and one eye in the IVB group showed an IOP increase of at least 5 mmHg compared to baseline (no eye exceeded an IOP of 25 mmHg). In contrast, this IOP threshold was exceeded in thirteen eyes in the IVT group; topical treatment was administered in four of these cases (when IOP > 24 mmHg), one of which required filtering surgery because the maximal topical treatment dose failed to control the IOP. 

After the follow-up period, cataract progression was observed in two eyes in the PRP group, in one eye in the IVB group (which was not significantly different from the PRP group) and in eight eyes in the IVT group (*P* = 0.029 when compared with the PRP eyes), but in no case cataract surgery was performed during the follow-up period. There were three cases of exudative peripheral retinal detachment after the laser treatment (one in the control group and two in the IVT group), which spontaneously resolved with topical nonsteroidal anti-inflammatory drops. One patient presented with acute anterior uveitis after the third bevacizumab injection; this was completely resolved with steroid drops. Another patient in the IVB group suffered a nonfatal myocardial infarction one month after the first bevacizumab injection. No more injections were administered in this patient.

Vitreous haemorrhage occurred in two eyes from the PRP group, which were excluded from the data because outcome measures could not be evaluated. Four eyes (one in the PRP group, two in the IVT group and one in the IVB group) were lost to followup because the patients could not attend follow-up visits for various reasons. Data from the remaining eyes were included in the analysis.

## 4. Discussion

The guidelines established by the ETDRS and DRS indicate the use of PRP in cases of proliferative diabetic retinopathy (PDR). However, when PDR coexists with macular oedema, the recommendations are less clear. It has been reported that PRP alone can lead to a gain of two or more ETDRS lines in 45% of patients with severe macular oedema and PDR [23]. However, delaying PRP can cause vision loss although it is known that PRP also worsens untreated macular oedema [24, 25].

To date, the present study is the first to compare the results of PRP alone to the combination of PRP and intravitreal triamcinolone or bevacizumab injections in naïve eyes.

The effects of 4 mg of triamcinolone in the eye was expected to persist for at least three months [26]. Because the effects of anti-VEGF agents in the eye are more short lived and because previous studies on other ocular diseases, such as age-related macular degeneration, suggested a loading dose of three injections [27], our study included three bevacizumab injections one month apart and a single dose of triamcinolone to maintain the effects of these drugs for the same length of time.

In this study, to reduce the risk of worsening macular oedema, PRP was applied in 3 sessions, one week apart). However, it has been recently reported that changes in CMT were not different in patients who were treated in a single session compared to those who were treated in four sessions that were one week apart [28]. 

The main goal of this study was to evaluate the regression of the new vessels (NVs). We considered an NV to have regressed when no leakage was observed during any phase of the angiogram. At the end of the 6-month follow-up period, PRP led to the regression of 27.5% of NVs, and the combinations of PRP plus IVT and PRP plus IVB induced a significantly higher regression percentage (74.4% and 76.8% of NVs, resp.). No significant differences between the IVT and IVB groups were observed at the end of the follow-up period.

Combined treatments also resulted in more effective control of the retinopathy; the severity of the retinopathy did not worsen in either of the injected groups compared to a worsening that occurred in 15% of the eyes treated with PRP alone. The severity of the retinopathy was reduced in 65% of the eyes that received PRP plus IVT and 79% of those that received PRP plus IBV, whereas only 53% of the eyes in the PRP group showed reductions in the severity of the retinopathy. The data also suggest that the higher the severity of the retinopathy, the more effective the combined treatments; however, the small sample size of this study does not provide adequate power to fully determine these differences, and this should be an area for further study.

Several studies have investigated the regression of new vessels after various treatment modalities, but the criteria used to define regression were not always consistent [17, 18]. Only the recent studies that evaluated the changes in the leakage area before and after treatment presented objective data [9, 14]. A reduction in the leakage area does not indicate a complete inactivation of the new vessels, and these less active vessels can still induce complications, such as recurrent vitreous haemorrhages or tractional retinal detachment. More restrictive criteria allow a better assessment of the results that are achieved with the various treatment modalities.  Table 4 summarizes the results reported by several studies and the regression criteria used in each. Note that the results obtained in our study are consistent with those previous reports (see the results section).

Changes in macular thickness in the PRP group during the follow-up period were similar to those reported in other studies. Choi et al. [29] reported a decrease in the macular thickness two months after PRP and focal laser treatment. In the present study, the thickness two months after PRP was similar to the baseline values. At 6 months after treatment, Bandello et al. [9] reported a 17% increase in the macular thickness compared to baseline in patients who underwent PRP and focal laser treatment, which is in contrast to the results in the present study of 8.2% worsening after PRP alone.

Intravitreal bevacizumab failed to reduce the macular thickness; furthermore, at the six-month follow-up visit, there was a 14.6% worsening of CMT compared to the baseline. These results are less impressive than those reported by Cho et al. in similar studies, in which a 12.4% reduction in CMT was observed three months after PRP + IVB + focal laser treatment [19, 30]. Focal laser therapy may be required to stabilise the maculae in cases of coexisting macular oedema and PDR if bevacizumab injections are planned.

On the other hand, intravitreal triamcinolone significantly reduced macular thickness one and two months after the injections, but CMT returned to baseline after six months. Our results are poorer than those published by other groups [9, 29, 31]. Because we obtained the 4 mg in 0.1 mL of triamcinolone directly from the vial without any filtering or purification process, the actual injected dose may be lower than this amount, as was previously reported [26]. Because the effect of the triamcinolone depends greatly on the dose injected [32], the actual injected dose in our study could have been lower than that used by other groups (no data on the actual injected doses are provided in these studies, except the Choi et al. study [29]). Similar to eyes treated with bevacizumab, eyes treated with PRP + focal laser therapy + IVT appear to be associated with better results, according to the literature [31]. 

Although no significant differences between the IVT and IVB groups were observed at the end of the follow-up period in the present study, a recent study showed that triamcinolone was more effective than bevacizumab at reducing, or at least maintaining, the macular thickness [30]. Unlike bevacizumab, triamcinolone is not limited to blocking VEGF. Triamcinolone has also been linked to reductions in other inflammatory cytokines, such as IL6, ICAM-1, MCP-1, and PDEF, which have all been implicated in the pathogenesis of macular oedema [33].

Both triamcinolone and bevacizumab failed to increase the visual acuity compared to PRP alone. BCVA remained stable in the different groups during the follow-up period, and no correlation with CMT was observed. These findings are similar to those reported for eyes that were treated with laser therapy alone [9, 29] and for eyes that were treated with bevacizumab [14, 19], but the results are poorer than those in eyes adjunctively treated with triamcinolone [9, 29, 31], as discussed above.

Panretinal photocoagulation does not result in reduced contrast sensitivity. The addition of bevacizumab also did not reduce the contrast sensitivity, but the combination of PRP plus IVT resulted in a reduced Pelli-Robson score at the six-month follow-up visit. This reduction, which was significant compared to baseline, was not significant compared to the PRP alone group and the IVB plus PRP.

Injections have been shown to be relatively safe. No complications related to the injections were observed in the present study, and apart from increased ocular pressure in the IVT group (which was controlled with topical treatment in all except in one case) and a case of acute anterior uveitis in one patient following the third bevacizumab injection, no other major ocular side effects were observed. One patient suffered a nonfatal myocardial infarction one month after the first intravitreal bevacizumab treatment. Although the timeline and presence of other risk factors suggest no relationship between the treatment and the heart attack, no subsequent injections were administered in this patient.

This nonblinded study has several limitations in addition to the small sample size. The IVB group was not randomly formed because the original study design considered only the PRP and PRP plus IVT groups. The increasing importance of bevacizumab in the treatment of multiple eye diseases compelled us to create this new group to investigate the role of bevacizumab in PDR treatment. 

A six-month follow-up period might be considered to be short, but we decided to analyse the results at this time point for several reasons. First, the effect of PRP is considered to be stable at six months after the procedure [16, 17, 34], and the effects of both intravitreal drugs were not expected to persist beyond this period. In addition, similar studies with a follow-up period of one year did not show changes in the visual acuity at the one-year follow-up visit compared to the 6-month follow-up visit [9, 31], and the macular thickness also remained stable [31]. Finally, because focal laser therapy was not administered before or during the follow-up period, the patients were reevaluated, and focal laser retreatments were considered at the six-month visit. 

In conclusion, adjunct intravitreal triamcinolone or bevacizumab treatment is relatively safe and leads to better control of retinopathy in patients with diabetic proliferative retinopathy. It appears that the more severe the retinopathy, the more effective the combination treatment. In cases of coexisting macular oedema and PDR, triamcinolone may be a better choice than bevacizumab because thickening of the macula was not observed in the IVT group. Based on the results in the present study and data from other reports, focal laser therapy at the time of the PRP seems to improve the results for both the IVT and the IVB groups.

The findings of this pilot study are an important addition to the literature, but they require confirmation in future clinical trials to determine the best therapeutic option for these patients.

## Figures and Tables

**Table 1:** Baseline clinical characteristics.

	PRP group *n* = 20	IVT group *n* = 20	IVB group *n* = 20	*P* value
Male : female	16 : 4	14 : 6	15 : 5	0.77
Age mean (SD)	55.10 (14.8)	55.28 (13.5)	53.77 (13.8)	0.936
Type of diabetes 1 : 2 (no. of eyes)	4 : 16	4 : 16	5 : 14	0.86
BCVA, mean no. of ETDRS letters (SD)	67.35 (22.5)	76.65 (9.99)	75.53 (18.99)	0.212
CMT mean (SD)	342.05 (118.69)	323.85 (157.33)	309.05 (89.84)	0.715
No. of new vessels	3.45 (2.01)	4.35 (3.32)	4.79 (4.36)	0.449
Mild PDR (no. of eyes)	5	9	3	—
Moderate PDR (no. of eyes)	10	6	9	—
High-risk PDR (no. of eyes)	3	5	6	—
Advanced high-risk PDR (no. of eyes)	2	0	2	—
BP (mmHg)	135/82	135/81	126/77	0.25/0.18
Glycaemia (mg/dL)	174.65	186.55	152.68	0.308
Cholesterol (mg/dL)	200.1	196.3	196.15	0.967
LDL-Cholest. (mg/dL)	133.26	125.72	126.88	0.86
HDL-Cholest. (mg/dL)	41.94	43.21	49.44	0.105
Triglycerides (mg/dL)	131.4	145.4	109.73	0.19
HbA1c	8.325	8.315	7.87	0.65

BCVA: best-corrected visual acuity; BP: blood pressure; Cholest: cholesterol; CMT: central macular thickness; dL: decilitres; ETDRS: early treatment diabetic retinopathy study; HbA1c: glycosylated haemoglobin; HDL: high-density lipoproteins; IVB: intravitreal bevacizumab; IVT: intravitreal triamcinolone; LDL: low-density lipoproteins; mg: milligrams; mmHg: millimetres of mercury; no.: number; PDR: proliferative diabetic retinopathy; PRP: panretinal photocoagulation; SD: standard deviation. ANOVA was used to compare data among the groups.

**Table 2:** Changes in the number of active new vessels (No. NV), best corrected visual acuity (BCVA), and central macular thickness (CMT) in the different groups before and after treatments, expressed as mean (SD).

	Baseline			1 month		2 months			6 months		
	No. NV	BCVA	CMT	BCVA	CMT	No. NV	BCVA	CMT	No. NV	BCVA	CMT
PRP group	3.45 (2.0)	67.35 (22.5)	342.05 (118.7)	64.25 (21.60)	374.75 (141.6)	2.28 (2.2)	65.68 (20.65)	347.75 (101.7)	2.5 (2.1)	64.88 (21.74)	370.2 (106.1)
				*P* = 0.183	*P* = 0.124	*P* = 0.049	*P* = 0.521	*P* = 0.402	*P* = 0.064	*P* = 0.055	*P* = 0.129
IVB group	4.79 (4.4)	75.53 (18.99)	309.05 (89.9)	74.88 (16.49)	313.94 (85.9)	0.05	75.32 (15.15)	314.58 (104.2)	1.11 (1)	74.74 (17.14)	354.3 (133.4)
				*P* = 0.623	*P* = 0.902	*P* = 0.001	*P* = 0.923	*P* = 0.671	*P* = 0.002	*P* = 0.628	*P* = 0.032
IVT group	4.35 (3.3)	76.65 (9.99)	323.09 (157.3)	74.47 (9.02)	258.79 (69.7)	1.25 (1.9)	74.75 (11.67)	279.75 (89.6)	1.11 (1.4)	72.72 (12.69)	310.9 (110.1)
				*P* = 0.278	*P* = 0.02	*P* = 0.001	*P* = 0.191	*P* = 0.134	*P* = 0.001	*P* = 0.011	*P* = 0.857

The Student's *t* test was used to compare the changes in outcomes to the baseline for the three groups. BCVA, best-corrected visual acuity; CMT, central macular thickness; No. NV: number of new vessels; IVB: intravitreal bevacizumab; IVT: intravitreal triamcinolone; PRP: panretinal photocoagulation; SD: standard deviation.

**Table 3:** Severity of diabetic retinopathy. Number of eyes per severity level before and after treatment in the three groups. Inactive PDR refers to eyes with complete regression of the new vessels after treatment.

	PRP group			IVT group			IVB group		
	Basal	2 months	6 months	Basal	2 months	6 months	Basal	2 months	6 months
Inactive PDR	—	5	3	—	10	9	—	16	6
Mild PDR	5	7	9	9	7	7	3	1	11
Moderate PDR	10	4	4	6	2	3	8	0	1
High-risk PDR	3	2	2	5	1	0	6	1	0
Advanced high-risk PDR	2	2	1	0	0	0	2	1	1

IVB: intravitreal bevacizumab; IVT: intravitreal triamcinolone; PDR: proliferative diabetic retinopathy; PRP: panretinal photocoagulation.

**Table 4:** Summary of the studies that evaluated the regression of new vessels in diabetic retinopathy after various treatment modalities.

Study	Type of study	Efficacy measure	Results
Doft and Blankenship [16]	Prospective study. 54 eyes included, with a six-month followup	Proportion of eyes that showed reduced levels of severity and had 3 or more risk factors	72% of these eyes remained stable three weeks after PRP 72% of these eyes remained stable at a six-month visit
Vander et al. [17]	Retrospective study. 59 eyes included, with a twelve-month followup	NVD moderate-severe: reduction of NVD in 1/3 of DA Mild NVD: complete regression of NVD	Regression was observed in 62% of these eyes six months after PRP
Reddy et al. [18]	Retrospective study. 294 eyes included, with a twelve-month followup	4 high-risk characteristics: reduction to 2 or less 2 high-risk characteristics: reduction to 1	Regression was observed in 77% of these eyes one year after PRP
Bandello et al. [9]	Prospective, comparative and randomised study. 9 eyes included per group, with a twelve-month followup	Reduction of the leakage area of the new vessels based on FA	Observations at three, six, and nine months revealed that 19%, 22%, and 33% reductions of the leakage area, respectively, occurred in the eyes that were treated with laser therapy Observations at one, six, and nine months revealed that 74%, 84%, and 86% reductions of the leakage area, respectively, occurred in the eyes that were treated with laser therapy and IVT
Zein et al. [10]	Prospective study. 35 eyes included with a nine-month followup, and data were compared retrospectively with medical records	Stabilisation: regression or no progression of the new vessels	100% of the eyes in the laser group at a nine-month visit 100% of eyes treated with the combination of laser and IVT
IBehi study [14]	Prospective, comparative and randomised study. 15 eyes included per group, with a six-month followup	Reduction of the leakage area of the new vessel based on FA	3.78%, 6.8%, and 11.2% in eyes treated with laser therapy (four, nine, and sixteen weeks, resp.) 94.5%, 93.99%, and 60% in eyes treated with laser therapy and IVB (four, nine, and sixteen weeks, resp.)
Cho et al. [19]	Prospective, comparative and randomised study. 19 eyes included per group, with a three-month followup	Worsening severity of the retinopathy	20% of eyes treated with laser therapy after three months 0% of eyes treated with laser and IVB after three months
Lopez-Lopez et al.	Prospective, comparative study. The first 40 eyes were randomised (1 : 1) to a PRP group or to an IVT group. The IVB group included 20 consecutive eyes that met the inclusion criteria. Six-month followup	Complete absence of leakage based on FA after treatment was applied	33% and 27.5% in eyes treated with laser therapy (two and six months, resp.) 71.2% and 74.4% in eyes treated with laser therapy and IVT (two and six months, resp.) 98.9% and 76.8% in eyes treated with laser therapy and IVB (two and six months, resp.)

DA: disc area; FA: fluorescein angiography; IVB: intravitreal bevacizumab; IVT: intravitreal triamcinolone; NVD: neovascularisation of the disc; PRP: panretinal photocoagulation.

## References

[1] Kempen J, O'Colmain B, Leske M (2004). The prevalence of diabetic retinopathy among adults in the United States. *Archives of Ophthalmology*.

[2] Klein R, Klein BEK, Moss SE (1984). The Wisconsin Epidemiologic Study of Diabetic Retinopathy. II. Prevalence and risk of diabetic retinopathy when age at diagnosis is less than 30 years. *Archives of Ophthalmology*.

[3] Klein R, Klein BEK, Moss SE (1984). The Wisconsin Epidemiologic Study of Diabetic Retinopathy. III. Prevalence and risk of diabetic retinopathy when age at diagnosis is 30 or more years. *Archives of Ophthalmology*.

[4] Adamis AP, Miller JW, Bernal MT (1994). Increased vascular endothelial growth factor levels in the vitreous of eyes with proliferative diabetic retinopathy. *American Journal of Ophthalmology*.

[5] Aiello LP, Avery RL, Arrigg PG (1994). Vascular endothelial growth factor in ocular fluid of patients with diabetic retinopathy and other retinal disorders. *New England Journal of Medicine*.

[6] Pe’er J, Shweiki D, Itin A, Hemo I, Gnessin H, Keshet E (1995). Hypoxia-induced expression of vascular endothelial growth factor by retinal cells is a common factor in neovascularizing ocular diseases. *Laboratory Investigation*.

[7] The Diabetic Retinopathy Study Research Group (1981). Photocoagulation treatment of proliferative diabetic retinopathy. Clinical application of diabetic retinopathy study (DRS) findings, DRS report number 8. *Ophthalmology*.

[8] Zacks DN, Johnson MW (2005). Combined intravitreal injection of triamcinolone acetonide and panretinal photocoagulation for concomitant diabetic macular edema and proliferative diabetic retinopathy. *Retina*.

[9] Bandello F, Polito A, Pognuz DR, Monaco P, Dimastrogiovanni A, Paissios J (2006). Triamcinolone as adjunctive treatment to laser panretinal photocoagulation for proliferative diabetic retinopathy. *Archives of Ophthalmology*.

[10] Zein WM, Noureddin BN, Jurdi FA, Schakal A, Bashshur ZF (2006). Panretinal photocoagulation and intravitreal triamcinolone acetonide for the management of proliferative diabetic retinopathy with macular edema. *Retina*.

[11] Spaide RF, Fisher YL (2006). Intravitreal bevacizumab (Avastin) treatment of proliferative diabetic retinopathy complicated by vitreous hemorrhage. *Retina*.

[12] Mason JO, Nixon PA, White MF (2006). Intravitreal injection of bevacizumab (Avastin) as adjunctive treatment of proliferative diabetic retinopathy. *American Journal of Ophthalmology*.

[13] Mirshahi A, Roohipoor R, Lashay A, Mohammadi SF, Abdoallahi A, Faghihi H (2008). Bevacizumab-augmented retinal laser photocoagulation in proliferative diabetic retinopathy: a randomized double-masked clinical trial. *European Journal of Ophthalmology*.

[14] Tonello M, Costa RA, Almeida FPP, Barbosa JC, Scott IU, Jorge R (2008). Panretinal photocoagulation versus PRP plus intravitreal bevacizumab for high-risk proliferative diabetic retinopathy (IBeHi study). *Acta Ophthalmologica*.

[15] Minnella AM, Savastano CM, Ziccardi L (2008). Intravitreal bevacizumab (Avastin) in proliferative diabetic retinopathy. *Acta Ophthalmologica*.

[16] Doft BH, Blankenship G (1984). Retinopathy risk factor regression after laser panretinal photocoagulation for proliferative diabetic retinopathy. *Ophthalmology*.

[17] Vander JF, Duker JS, Benson WE, Brown GC, McNamara JA, Rosenstein RB (1991). Long-term stability and visual outcome after favorable initial response of proliferative diabetic retinopathy to panretinal photocoagulation. *Ophthalmology*.

[18] Reddy VM, Zamora RL, Olk RJ (1995). Quantitation of retinal ablation in proliferative diabetic retinopathy. *American Journal of Ophthalmology*.

[19] Cho WB, Oh SB, Moon JW, Kim HC (2009). Panretinal photocoagulation combined with intravitreal bevacizumab in high-risk proliferative diabetic retinopathy. *Retina*.

[20] Early Treatment Diabetic Retinopathy Study Research Group (1991). Fundus photographic risk factors for progression of diabetic retinopathy: ETDRS report number 12. *Ophthalmology*.

[21] (1991). Early Treatment Diabetic Retinopathy Study design and baseline patient characteristics: ETDRS report number 7. *Ophthalmology*.

[22] Chylack LT, Wolfe JK, Singer DM (1993). The Lens Opacities Classification System III. The Longitudinal Study of Cataract Study Group. *Archives of Ophthalmology*.

[23] Gardner TW, Eller AW, Friberg TR (1991). Reduction of severe macular edema in eyes with poor vision after panretinal photocoagulation for proliferative diabetetic retinopathy. *Graefe’s Archive for Clinical and Experimental Ophthalmology*.

[24] McDonald HR, Schatz H (1985). Macular edema following panretinal photocoagulation. *Retina*.

[25] McDonald HR, Schatz H (1985). Visual loss following panretinal photocoagulation for proliferative diabetic retinopathy. *Ophthalmology*.

[26] Spandau UHM, Derse M, Schmitz-Valckenberg P, Papoulis C, Jonas JB (2005). Dosage dependency of intravitreal triamcinolone acetonide as treatment for diabetic macular oedema. *British Journal of Ophthalmology*.

[27] Lalwani GA, Rosenfeld PJ, Fung AE (2009). A variable-dosing regimen with intravitreal ranibizumab for neovascular age-related macular degeneration: year 2 of the PrONTO Study. *American Journal of Ophthalmology*.

[28] Brucker AJ, Qin H, Antoszyk AN (2009). Observational study of the development of diabetic macular edema following panretinal (scatter) photocoagulation given in 1 or 4 sittings. *Archives of Ophthalmology*.

[29] Choi KS, Chung JK, Lim SH (2007). Laser photocoagulation combined with intravitreal triamcinolone acetonide injection in proliferative diabetic retinopathy with macular edema. *Korean Journal of Ophthalmology*.

[30] Cho WB, Moon JW, Kim HC (2010). Intravitreal triamcinolone and bevacizumab as adjunctive treatments to panretinal photocoagulation in diabetic retinopathy. *British Journal of Ophthalmology*.

[31] Maia OO, Takahashi BS, Costa RA, Scott IU, Takahashi WY (2009). Combined laser and intravitreal triamcinolone for proliferative diabetic retinopathy and macular edema: one-year results of a randomized clinical trial. *American Journal of Ophthalmology*.

[32] Kim JE, Pollack JS, Miller DG, Mittra RA, Spaide RF (2008). ISIS-DME: a prospective, randomized, dose-escalation intravitreal steroid injection study for refractory diabetic macular edema. *Retina*.

[33] Funatsu H, Noma H, Mimura T, Eguchi S, Hori S (2009). Association of vitreous inflammatory factors with diabetic macular edema. *Ophthalmology*.

[34] Blankenship GW (1991). Fifteen-year argon laser and xenon photocoagulation results of Bascom Palmer Eye Institute’s patients participating in the diabetic retinopathy study. *Ophthalmology*.

